# Sepsis and Hemocyte Loss in Honey Bees (*Apis mellifera*) Infected with *Serratia marcescens *Strain Sicaria

**DOI:** 10.1371/journal.pone.0167752

**Published:** 2016-12-21

**Authors:** Nancy L. Burritt, Nicole J. Foss, Eric C. Neeno-Eckwall, James O. Church, Anna M. Hilger, Jacob A. Hildebrand, David M. Warshauer, Nicole T. Perna, James B. Burritt

**Affiliations:** 1 Department of Biology, University of Wisconsin-Stout, Menomonie, WI, United States of America; 2 Biotechnology Center, University of Wisconsin-Madison, Madison, WI, United States of America; 3 Department of Biology, LaSalle University, Philadelphia, PA, United States of America; 4 Wisconsin State Laboratory of Hygiene, University of Wisconsin-Madison, Madison, WI, United States of America; 5 Genome Center of Wisconsin, University of Wisconsin-Madison, Madison, WI, United States of America; Pusan National University, REPUBLIC OF KOREA

## Abstract

Global loss of honey bee colonies is threatening the human food supply. Diverse pathogens reduce honey bee hardiness needed to sustain colonies, especially in winter. We isolated a free-living Gram negative bacillus from hemolymph of worker honey bees (*Apis mellifera*) found separated from winter clusters. In some hives, greater than 90% of the dying bees detached from the winter cluster were found to contain this bacterium in their hemolymph. Throughout the year, the same organism was rarely found in bees engaged in normal hive activities, but was detected in about half of *Varroa destructor* mites obtained from colonies that housed the septic bees. Flow cytometry of hemolymph from septic bees showed a significant reduction of plasmatocytes and other types of hemocytes. Interpretation of the16S rRNA sequence of the bacterium indicated that it belongs to the *Serratia* genus of Gram-negative Gammaproteobacteria, which has not previously been implicated as a pathogen of adult honey bees. Complete genome sequence analysis of the bacterium supported its classification as a novel strain of *Serratia marcescens*, which was designated as *S*. *marcescens* strain sicaria (Ss1). When compared with other strains of *S*. *marcescens*, Ss1 demonstrated several phenotypic and genetic differences, including 65 genes not previously found in other *Serratia* genomes. Some of the unique genes we identified in Ss1 were related to those from bacterial insect pathogens and commensals. Recovery of this organism extends a complex pathosphere of agents which may contribute to failure of honey bee colonies.

## Introduction

### Honey Bee Decline

Humans increasingly rely on pollinators for food production. In spite of the expanding need for pollination and gene flow in flowering crop plants, mortality rates in managed honey bee hives are threatening many beekeeping operations [1–3]. The Bee Informed Partnership (http://beeinformed.org) reported a loss of 44% of US hives in 2015–2016, a 3.5% increase from the previous year, and a 12.5% increase when compared to 2013–2014. These data further support the hive loss rates that have exceeded acceptable levels for more than one decade, despite a global effort to reduce bee diseases.

### Overview on Infectious Agents of Honey Bees

Impacts on honey bee health are often regarded to be multifactorial, potentially involving exposure to insecticides [4–6], erosion of the bee’s immune system [7–9], and a complex set of infectious agents [10,11]. We now recognize a spectrum of microbial pathogens of honey bees, including approximately 20 different RNA viruses, four bacteria, three fungi, and several protozoan parasites [10,12–14]. Multicellular parasites and pests further complicate the bee’s ability to sustain the intricate activities of the colony, especially during winter.

*Varroa destructor* mites parasitize honey bees and serve as a vector for transmission of several viruses [15]. Many current strategies for managing colony health therefore target *V*. *destructor* populations. However, growing concern over acaracide resistance in mites and increasingly heavy-handed chemotherapeutic intervention in beekeeping practices [16,17] underscore the need for more sustainable strategies in hive management.

### Bacterial Diseases of Honey Bees

Bacterial infections in honey bees generally manifest as brood diseases [10], primarily involving American and European foulbrood. American foulbrood is caused by the endospore-forming *Paenibacillus larvae* bacterium and is often regarded as the most destructive of the reported bacterial bee brood diseases. European foulbrood is caused by the nonsporulating bacterium, *Melissococcus plutonius* [18]. Symptoms of these two types of foulbrood are similar and both are treated with some success using polycyclic antibiotics administered through supplemental feeding. *Serratia marcescens* was reported on one occasion to cause brood disease in honey bees [19]. However, to the best of our knowledge, *S*. *marcescens* has not previously been reported as a circulating pathogen of adult bees.

Examples of bacterial diseases of adult honey bees include powdery scale (caused by *Bacillus pulvifaciens*) and May disease (resulting from two species of the *Spiroplasma* genus) [10]. May disease generally occurs in mid-spring months during extended periods of cool and wet weather. *Spiroplasma melliferum* has recently been implicated as the primary cause of May disease in US bee colonies [20]. Other bacterial isolates have been investigated as possible honey bee pathogens, though none have been implicated as a primary cause of disease in adult bees.

### Our Findings

We report bacterial infection in live adult worker and drone honey bees found immobilized and separated from active hive members. We also isolated this bacterium from dead bees obtained from 24 of 33 winterkilled hives. In addition, this organism was recovered from a subset of *V*. *destructor* examined in area hives and in mites from a shipment of packaged bees delivered in April 2016 from a bee supply company outside the region of our study. Recovery of the organism from mites suggests a possible transmission mechanism for this bacterium among honey bees and hives.

Whole-genome nucleotide sequence analysis [21,22] confirmed this bacterium is a novel strain of *Serratia marcescens*. We named this organism *S*. *marcescens* strain sicaria (sicarius, Latin, meaning assassin), due to its recovery from diseased and dead honey bees. Herein, we refer to this strain of *S*. *marcescens* as Ss1.

Our study indicates infection of bees by Ss1 is associated with reduced numbers of host-defensive hemocytes as well as sepsis and death. These findings suggest this infection may truncate the bee’s life span, and therefore compromise the ability of hives to sustain the critical population needed to survive the winter. However, the ecological relationship between honey bees, *V*. *destructor*, and Ss1, and the consequences of their interactions in hive homeostasis, will require further study.

## Results

We used three criteria for defining our sample groups of live bees. The first criterion for classification was asymptomatic versus symptomatic bees. The common findings of symptomatic drones and symptomatic workers were that both groups demonstrated loss of normal movement and were found to be separated from the active hive members. Symptomatic workers were found living but immobile on the top of the inner cover during December and January. Symptomatic drones were unable to fly during warm months. The second criterion was infected versus uninfected bees, as determined by culture results. The third was worker bees versus drones, as determined by gender.

We therefore examined the following four experimental groups of live bees based on their characteristics and gender: 1) symptomatic infected workers (SIW) were separated from the winter cluster and showed Ss1 in hemolymph cultures, 2) asymptomatic infected workers (AIW) were identified among active hive members because of Ss1 in hemolymph cultures, 3) asymptomatic uninfected workers (AUW) were randomly collected among active hive members and showed no sign of Ss1 in hemolymph culture, and 4) symptomatic infected drones (SID) were found separated from hive members during warm months and showed hemolymph cultures positive for Ss1. We also cultured hemocoel extracts of dead bees obtained from winterkilled hives, external surfaces of normal and infected bees, and freshly-voided fecal material from bees in early spring.

A total of 3,219 honey bees and 1,259 *V*. *destructor* mites were collected for analyses between December 2014 and September 2016 from 91 hives in west central Wisconsin and eastern Minnesota, USA. From the hives examined, 33 hives were sampled for mites; from which 25 (76%) hives contained mites with cultures positive for Ss1. Of the same 91 hives, 66 were sampled for bees; from which 32 (48%) produced cultures positive for Ss1. In addition, 33 of the 91 hives were found winterkilled between January 1^st^ and April 1^st^, and were therefore used as a source of dead bees. When examining winterkilled hives, 24 of 33 (73%) of the winterkilled hives contained bees with positive cultures for Ss1.

### Isolation of Ss1 from Septic Honey Bees and *V*. *destructor*

**Fig 1:**
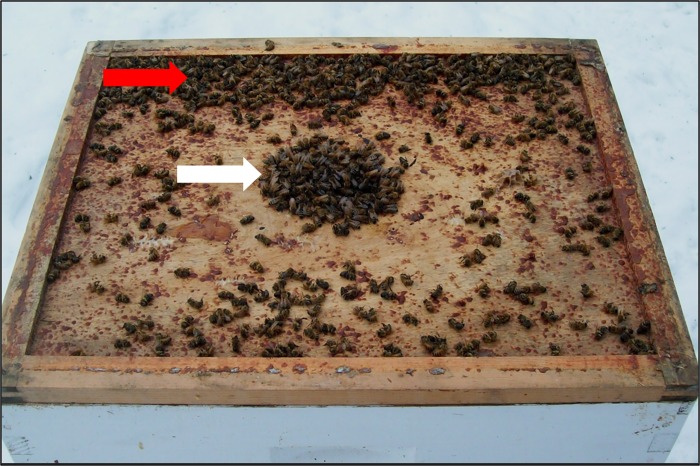
Normal and diseased bees associated with the winter bee cluster. Active bees (white arrow) as well as immobilized living and dead bees (red arrow) were seen when the outdoor temperature was -7°C. These bees were located on the inner cover of a representative Langstroth hive in Wisconsin. The active bees were contiguous with the winter cluster that extended through the 27.2 cm^2^ rectangular opening in the inner cover. The immobilized living and dead bees were marginalized from the cluster on top of the inner hive cover as shown. The immobilized bees included living SIW that revealed high densities of Ss1 when cultured on LB agar as described in the Methods.

When outdoor temperatures drop seasonally, honey bees naturally undergo cluster formation to conserve heat and sustain hive activity [23]. As such, honey bees have the unusual ability among insects to sustain a temperature-controlled environment during winter. At this time, we observed living septic bees separated from the winter cluster. Fig 1 shows the top of an exposed inner cover from a representative Langstroth hive from western Wisconsin in January 2016, when the outdoor temperature was -7°C. The image shows active worker bees (white arrow), which comprised part of the winter cluster, protruding through a 27.2 cm^2^ rectangular opening in the inner cover. Unfrozen immobilized living and dead bees (red arrow) were found detached from the cluster.

When the immobilized bees were warmed to 22°C for 4 minutes, a subset resumed moribund activity. Hemolymph was collected from those bees, plated on LB agar (and was sometimes also subjected to hemocyte profiling), and cultures were incubated at 22°C for three days. These cultures typically revealed heavy populations of Ss1, though no significant number of other microbes was found by the culture system we utilized. When collecting samples of bees immobilized in this way, 90.4% (+/- 3.3%, n = 8 samples from different hives) of those revived after warming produced positive cultures for Ss1, and therefore met our definition of SIW.

**Fig 2:**
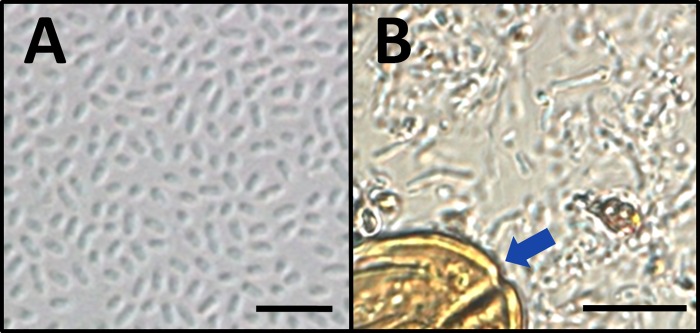
Photomicrographs representing direct wet mount images of hemolymph from SIW. Panel A shows a hemolymph sample obtained from one SIW, including a high density of bacteria with uniform morphology (Ss1), and an absence of pollen grains. Panel B shows a wet mount of hemolymph that was obtained from a different SIW, revealing mixed bacterial morphologies and occasional pollen grains (blue arrow). Cultures prepared from SIW collectively grew >1.0e9 cfu/ml Ss1. The scale bar represents 5 μm in Panel A and10 μm in Panel B.

**Fig 3:**
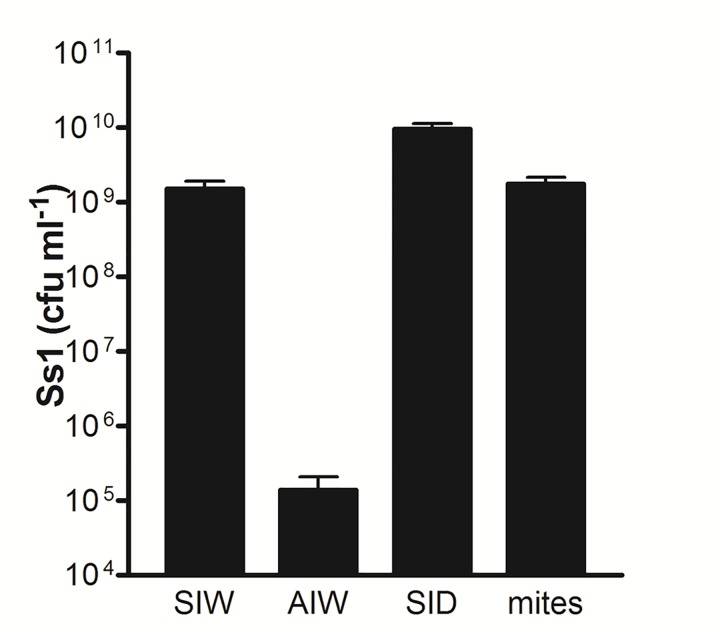
Concentrations of Ss1 obtained from bees and *V*. *destructor* examined in the study. Hemolymph from SIW, AIW, and SID experimental groups of living bees was collected and assayed by serial dilution and aerobic culture for Ss1 on LB agar. For individual mites (*V*. *destructor*), the value represents the density of bacteria in the 3 μl water extract prepared as described in the Methods. The data represent n = 14 for AIW, and n = 16 for SIW and SID. One-way ANOVA confirmed the concentration of Ss1 in AIW was significantly lower (P<0.05) when compared with both SIW and SID.

Direct microscopy of hemolymph from SIW showed a dearth of all hemocyte types among high concentrations of bacteria. In one subset of the SIW, the hemolymph was densely populated by bacteria with a single uniform rod-shaped morphology (consistent with Ss1), as shown by the wet mount slide preparation in Fig 2A. In other SIW, hemolymph contained mixed bacterial morphologies and occasional pollen grains (blue arrow, Fig 2B). When sampling SIW that produced hemolymph containing pollen, we frequently found turbid yellow fluid (hemolymph from normal bees is clear) liberated immediately upon puncture of the abdominal cuticle. This observation suggested that mixing of the hemolymph and digestive contents had occurred prior to sample collection. Bacterial culture of hemolymph represented by both Fig 2A and 2B revealed growth of a single type of aerobic Gram negative rod (Ss1). This organism was present in SIW at a concentration of 1.5 x 10e9, +/- 3.9 x 10e8 colony forming units (cfu)/ml, n = 16, as shown in Fig 3. At outdoor temperatures above 16°C, SIW were not observed either inside or outside hives.

Hemolymph from a subset of SIW contained pollen (as shown in Fig 2B) and appeared to include a diverse population of microbes originating from the digestive tract. However, bacterial cultures of those samples produced only Ss1. We therefore tested the possibility that most intestinal microbes of the honey bee were not represented in our culture results. Uninfected living worker bees were collected, chilled on ice, and microbes from their digestive tracts were obtained and diluted in sterile water. The microbial densities in the samples prepared from individual bees were quantified by hemocytometer at 1.1 x10e10 microbes/ml +/- 2.2 x10e9 microbes/ml, n = 11. When those same samples were inoculated to LB agar plates and incubated for three days at 22°C (as we did while cultivating Ss1), the concentration of microbes was significantly lower (1.6 x10e3 cfu/ml +/- 9.5 x10e2 cfu/ml, n = 11, P = 0.0005). We therefore expect that the normal flora in honeybees is typically populated by organisms with growth requirements (including capnophilic and anaerobic conditions) that were not provided by our culture system [24,25].

We also examined worker bees for evidence of Ss1 carried in sources other than hemolymph. Surface cultures of normal and septic bees did not reveal Ss1. In addition, we cultured 128 samples of fecal material produced by bees as they emerged during early spring from hive clusters that also produced SIW. Those cultures also failed to reveal any evidence of Ss1. We concluded that Ss1 was not generally present on the surface of septic bees, or in the digestive tracts of bees healthy enough to fly from hives during early spring. However, we could not exclude the possibility that Ss1 is carried in the digestive tracts of SIW.

Between June 23^rd^ and December 1^st^ in 2015, 19 of 701 (<3%) active bees selected randomly from within hives revealed Ss1 in hemolymph at a comparatively low concentration (1.4 x 10e5 +/- 6.7 x 10e4 cfu/ml, n = 14 for culture-positive bees, Fig 3). The concentration of Ss1 in these AIW was significantly less (P<0.05) than the corresponding concentration in both SIW and SID. By contrast, AUW were involved in normal activity and did not show any evidence of Ss1 in hemolymph cultures.

**Fig 4:**
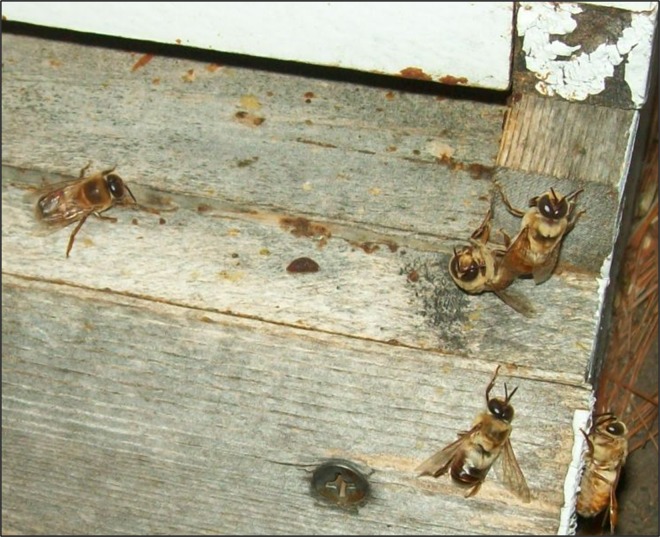
Drone honey bees showing symptoms of Ss1 infection. Drone honey bees that were unable to fly could be found attached to the hive near the entrance or crawling on the ground in front of the hive. Bacterial culture of the hemolymph from these drones revealed Ss1 in high concentration (9.5 x 10e9 +/-1.9 x 10e9 cfu/ml, n = 16). Unlike SIW, these SID were observed intermittently during warm seasons (>16°C).

SID were also recovered from hives that produced AIW and SIW. In contrast to the SIW which we found only when the outside temperature was below 4°C, the SID were observed during warm spring and summer months when outdoor temperatures were above 16°C. The SID were unable to fly and were often found clinging to the outside of the hive near the entrance (Fig 4), or crawling on the ground in front of hives. Compared with other sample groups, SID showed presence of Ss1 in the highest concentration (9.5 x 10e9 +/-1.9 x 10e9 cfu/ml, n = 16, Fig 3).

We verified that active bees generally do not contain Ss1 in their hemolymph. The active worker bees clustering in winter (represented by the white arrow in Fig 1) were sampled, and when their hemolymph was cultured, were free of infection by Ss1. Those bees constituted the AUW group. To show that active drones were not infected with Ss1, we installed queen excluders at hive entrances during mid-afternoon in warm weather when bees were flying. Workers returning to the hive passed through the excluder, but returning drones accumulated on the outside of the excluder where they were collected for culture analysis. Upon culturing hemolymph from 57 drones returning to the hive, none were found to be infected with Ss1, while 35 of 39 drones found on the ground and unable to fly contained Ss1 in their hemolymph.

From April to December 2015, Ss1 was also recovered from 51.2% (+/- 7.1%, n = 12) of *V*. *destructor* obtained from 25 of 33 hives in our region. The concentration of Ss1 in the 3μl mite extracts was 1.7e9 cfu/ml +/-4.2 x 10e8 cfu/ml, n = 16 (Fig 3), corresponding to 5.2 x 10e6 +/-1.3 x 10e6 cfu/mite. This degree of organism burden is consistent with experimental loading of a different strain of *S*. *marcescens* in the related ectoparasite, *Varroa jacobsoni* [26].

### Bacterial Culture of Bees from Winterkilled Hives

To determine whether Ss1 could be associated with winterkill of honey bee colonies, dead bees were obtained from recently-failed hives from December to April in both 2015 and 2016. Cultures prepared from dead bees collected from 24 of 33 winterkilled hives produced Ss1. The concentration of Ss1 from hemocoel extracts of bee carcasses prepared as described in the Methods was 1.2 x 10e10 +/-4.7 x 10e9 cfu/ml, n = 10. We observed that dead bees obtained from a diminished frozen cluster after hive failure were less likely to show evidence of the Ss1 than bees marginalized outside the cluster. We assume frozen cluster bees avoided infection by Ss1, but died of cold and starvation.

### 16S rRNA Sequence Analysis

Using the nucleotide BLAST function on the NCBI website (http://blast.ncbi.nlm.nih.gov/Blast.cgi), the 1541 base-pair segment of DNA corresponding to the 16S rRNA sequence of Ss1 (S1 Fig) showed a 99–100% match to the corresponding region in several different strains of *S*. *marcescens*. Although sufficient to identify our isolate as *S*. *marcescens*, this analysis did not provide information to explain the phenotypic differences of Ss1 when compared with other strains of *S*. *marcescens*, including the *S*. *marcescens* ATCC 13880 type strain. Therefore, the entire genome of Ss1 was sequenced (and deposited in Genbank under the accession number MEDA00000000) and compared with other organisms as described below. The whole-genome sequence of Ss1 should enable others to examine this bacterium for unique genetic elements that may contribute to its involvement with honey bees and *V*. *destructor*

### Whole-genome Taxonomic Classification of Ss1

Integrated comparative genomic classification of Ss1 was carried out using three independent taxonomic genetic methods [22]. Each approach utilized a nucleotide classification strategy with practical advantages when compared with DNA-DNA hybridization (DDH) and multiphasic phenotypic characterization [27,28]. Average nucleotide identity (ANI), average amino acid identity (AAI), and genome BLAST distance phylogeny [29] comparisons were applied to Ss1 for taxonomic evaluation, using other sequenced organisms for reference.

These approaches showed similar genetic distances regarding the phylogeny of Ss1, with overall agreement in maximum likelihood tree geographical structure. The percent identity scores of the Ss1 strain compared to the *S*. *marcescens* type strain are 94.90% for ANI and 96.38% for AAI. Identity scores for both ANI and AAI comparisons of Ss1 to 41 other *Serratia* genomes are included (S1 Dataset, Identity Scores). The Genome-to-Genome Distance Calculator [29] provided an estimated DDH value of 62.3%, leading to a probability of nearly 60% that the DDH is greater than 70%, which is commonly considered to be the cutoff between species [30]. This estimate, along with the ANI value of nearly 95%, puts Ss1 at the species boundary with the *S*. *marcescens* type strain. We selected AAI to compare the set of sequenced *Serratia* genomes because the nucleic acid identity values can be as low as 80% or less between different species, values for which AAI has greater accuracy in estimating bacterial diversity.

**Fig 5:**
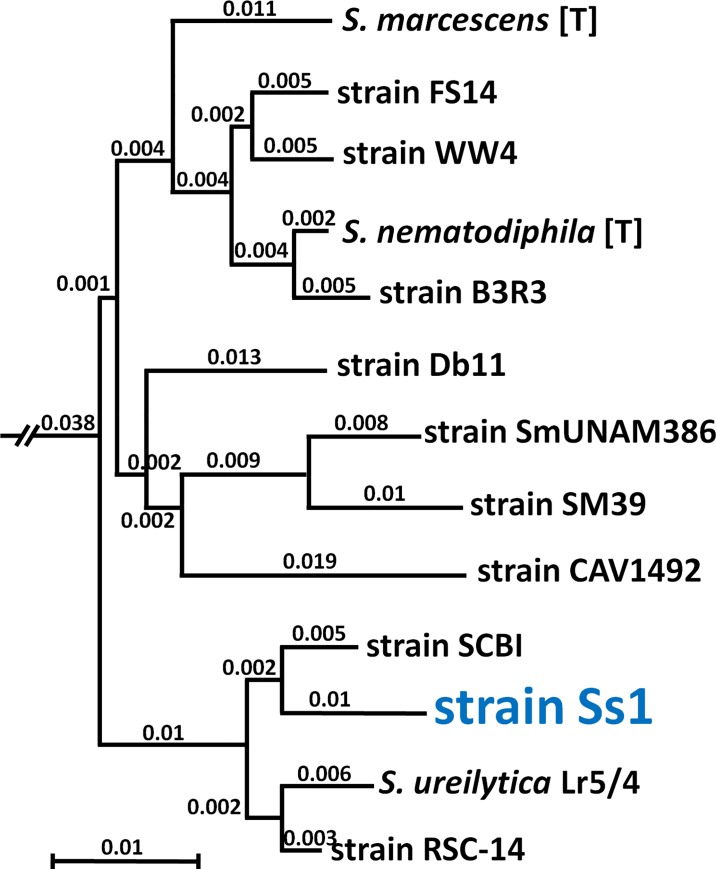
Dendrogram based on Average Amino Acid Identity (AAI) when comparing the whole-genome of Ss1 to 12 other members of the *Serratia* genus. The maximum likelihood tree based on AAI analysis illustrates hierarchical clustering that reveals a well-defined group of 13 organisms that constitutes the *S*. *marcescens* clade. Ss1 exists within this taxonomic cluster and shows greater than 96% similarity to the *S*. *marcescens* ATCC 13880 type strain based on AAI comparison. Branch lengths are displayed at the nodes, and the distance scale is shown at the base of the dendrogram.

The result of the AAI comparison is a phylogenetic dendrogram (Fig 5) that includes Ss1 with ten other complete genomes from strains of *S*. *marcescens* (including the type strain), and two genomes of other *Serratia* species. Interestingly, the closest genome match (98.49% similarity, as shown by AAI identity scores listed in the S1 Dataset) of Ss1 is to *Serratia* sp SCBI [31,32], a pathogen of the lepidopteran *Galleria mellonella* (honey comb moth), which is a pest of honey bee colonies. The complete dendrogram that includes the phylogenetic relationships of all 42 *Serratia* genomes based on AAI analysis is also provided as an unrooted phylogenetic tree (S2 Fig), and their distance scale values are shown in S1 Dataset.

We used the OrthoMCL [33] dataset to identify features in Ss1 that are not found in the other 41 *Serratia* genomes used to construct the phylogenetic trees shown in Fig 5 and S2 Fig. This information revealed 221 coding sequence (CDS) features that were unique to Ss1. A BLAST analysis of those sequences to the nonredundant (nr) database at NCBI was done to provide insight into function and origin of the deduced proteins, as shown (S2 Dataset, Ss1-specific Features). After culling the list for likely pseudogenes (including a length ratio of <0.5 relative to the GenBank sequence) and protein sequences shorter than 55 amino acids, 65 (29%) of the sequences were found to have potential origins in bacterial genera other than *Serratia*. Twelve of those genes showed similarity to bacterial symbionts of insects including *Plautia stali* (brown-winged green bug), *Frankliniella occidentalis* (western flower thrip), and *Nilaparvata lugens* (brown planthopper). Five others (showing no BLAST hit) did not reveal a match to any known sequences in the NCBI nr database, potentially representing novel genes.

### Biochemical Characterization of Ss1

**Fig 6:**
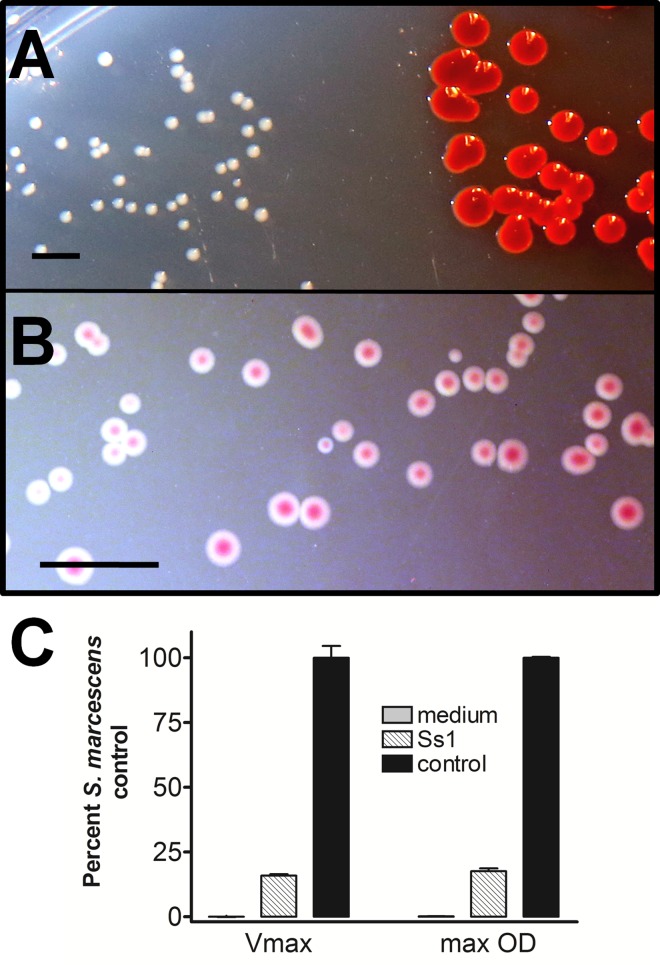
Colony phenotype characterization of Ss1. A) Side-by-side comparison identifies the smaller nonpigmented colonies of Ss1 (left) relative to the red-pigmented *S*. *marcescens* #361 control colonies (right) after 72 hours incubation at 22°C on LB agar. B) Following a five day incubation on MacConkey agar at 22°C, well-isolated colonies of Ss1 were about 1.5 mm in diameter, and when illuminated from below, showed burgundy-colored centers. C) Liquid growth characteristics of Ss1 (striped bars) were compared to the *S*. *marcescens* control strain (black bars), with reference to the growth medium alone (shaded bars). Values are expressed as a percentage of the control strain. Both the maximum growth rate (Vmax) and the maximum optical density at 600 nm (OD) of Ss1 showed significant (P<0.05) reduction when compared with the control strain. Liquid growth dynamic analyses were performed on three separate days with similar results. The scale bar in Figs A and B each represent 5 mm.

Despite the similarity in the genome sequence comparing Ss1 to other strains of *S*. *marcescens*, several phenotype and biochemical results of Ss1 are not typical for the characterized strains. For example, LB agar medium inoculated with both Ss1 and *S*. *marcescens* #361 and incubated for 72 hours at 22^°^C (as described in the Methods) reveals obvious colony appearance differences. Ss1 produced small white colonies (Fig 6A, left side of image) relative to the larger red-pigmented *S*. *marcescens* #361 (right side of the image). Following five days incubation at 22°C on MacConkey agar, well-isolated colonies of Ss1 were about 1.5 mm in diameter. When illuminated from beneath the culture medium with an artificial light, the well-isolated Ss1 colonies on MacConkey agar demonstrated centers with a burgundy color (Fig 6B). This color did not extend into the agar, and was therefore interpreted as pigment production within the colony, and not color from the pH indicator in the medium. Ss1 did not demonstrate several expected biochemical reactions typical for *S*. *marcescens*, including lysine decarboxylase, gelatinase, acetyl methyl carbinol production, citrate utilization, or esculin hydrolysis. A comprehensive set of biochemical analyses was performed on Ss1 and results were compared to those for typical laboratory isolates of *S*. *marcescens* [34] (Table 1).

**Table 1:** Biochemical reactions of Ss1 compared with common strains of *S*. *marcescens* ^*^.

Test	Ss1	*S*. *marcescens* (%)
2-nitrophenyl β-D-galactopyranoside	+	95
arginine dihydrolase	-	0
lysine decarboxylase	-	99
ornithine decarboxylase	+	99
citrate utilization	-	98
hydrogen sulfide production	-	0
Christensen urease	+	15
tryptophan deaminase	-	NA
indole	-	1
acetyl-methyl-carbinol (Voges-Proskauer)^#^	+	98
gelatinase	-	90
D-glucose	+	100
D-mannitol	+	99
myo-inositol	-	75
D-sorbitol	+	99
L-rhamnose	-	0
D-sucrose	+	99
melibiose	-	0
amygdalin	-	NA
L-arabinose	-	0
lactose	-	2
adonitol	+	40
maltose	+	96
trehalose	+	99
cellobiose	-	5
glycerol	+	95
raffinose	+	2
dulcitol	-	0
mucate	-	0
D-xylose	+	7
Jordan’s tartrate	+	75
methyl red	+	20
esculin hydrolysis	-	95
DNase	+	98

All reactions were incubated at 28°C except DNase (22°C).

*Percentage of *S*. *marcescens* showing positive results as reported [34].

^#^The Voges-Proskauer test was carried out in tube media.

NA = information not available.

One biochemical test Ss1 shares with most strains of *S*. *marcescens* is DNase activity at 22°C (S3 Fig). On DNase agar, Ss1 appears as a nonpigmented colony with clearing of blue color from the medium, which was evident even for a single isolated colony. This reaction serves as a useful screening tool to differentiate Ss1 from other bacteria sometimes encountered when sampling honey bees and *V*. *destructor*.

Colony size differences between Ss1 and the Presque Isle Culture #361 strain of *S*. *marcescens* suggest different growth dynamics. Replication characteristics of these organisms were therefore compared in liquid LB medium as described in the Methods. The most rapid growth (Vmax) for the control *S*. *marcescens* was found to occur between 300 and 333 minutes of incubation. This time frame was used as a reference to compare the growth rate for these organisms. The final turbidity of each culture was also recorded at 20 hours. The growth rate and final turbidity of Ss1 were then expressed as a percentage of the *S*. *marcescens* control (Fig 6C). Both indices were found to be significantly reduced (P<0.05) in Ss1, representing less than 18% of those for the control strain.

### Hemocyte Profiling

**Fig 7:**
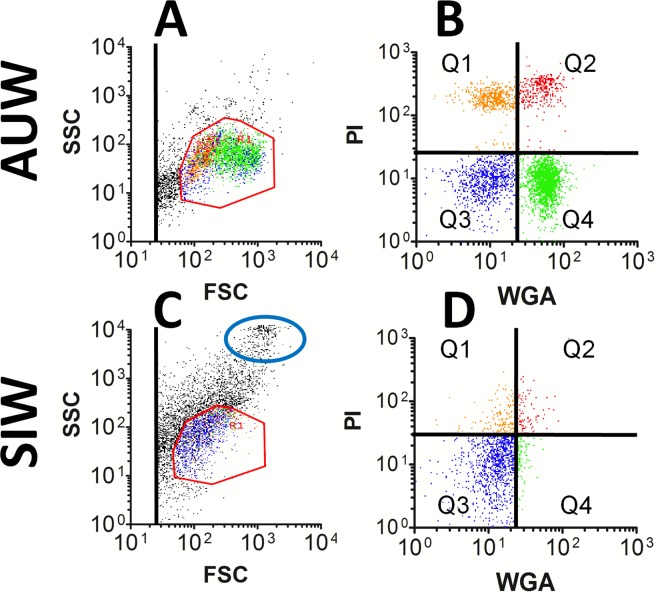
Hemocyte profiling by flow cytometry comparing normal and Ss1-infected bees. Hemolymph was collected from bees and probed with WGA-FITC and PI as described in the Methods. Data are shown for hemolymph particulates representing one AUW (A and B) and one SIW (C and D). FSC versus SSC identify physical parameters of the hemolymph particulates as shown in A and C, while green and red fluorescence values of the particulates included only in the red octagon gate are shown in B and D. SIW suggest a significant reduction in the abundance of circulating hemocytes represented in quadrants Q1, Q2, and Q4, but not in microparticles appearing in Q3. In SIW, the FSC versus SSC plots also indicate an increased number of large particulates (blue oval), consistent with the presence of pollen grains.

Direct microscopic analysis of honey bee hemolymph suggested hemocyte populations were reduced in SIW. This observation indicated Ss1 could be destroying hemocytes and thus suppressing cell-mediated immunity in infected bees. To further examine this possibility, hemocyte profiling by flow cytometry was used to compare the hemolymph particulates in normal and infected worker bees. Hemolymph particulates of single bees representative of AUW (Fig 7A and 7B) and SIW (Fig 7C and 7D) are shown. These plots display forward scatter (FSC) versus side scatter (SSC) parameters and also represent wheat germ agglutinin (WGA)-FITC versus propidium iodide (PI) fluorescence. A region including all hemocyte types [35] is defined in the FSC versus SSC scatter profiles (by the red octagon in Fig 7A and 7C) to focus fluorescence analysis on those cell types. Different colors were used to represent the particle types in the hemolymph in both the light scatter (Fig 7A and 7C) and fluorescence (Fig 7B and 7D) plots: orange and red dots represent permeabilized hemocytes showing low and high WGA-FITC binding, respectively, blue is used to display microparticles, and green identifies plasmatocytes.

We previously reported hemocytes in normal honey bees exist in two types of permeabilized cells (appearing in quadrants Q1 and Q2) and one type of unpermeabilized plasmatocyte (in quadrant Q4), as shown in Fig 7B [35]. When compared with AUW, the SIW showed reduction in all three hemocyte subsets identified by flow cytometry. This result corroborated our direct microscopy of hemolymph, indicating circulating hemocyte populations were reduced in SIW. Our flow cytometry data of SIW often revealed a prevalent population of large particles in hemolymph (outlined by the blue oval in Fig 7C), which was generally absent in normal bees. We verified this subset of large particles predominantly represents pollen grains, which was also consistent with our direct microscopy of hemolymph. Flow cytometry results supported the presence of pollen in hemolymph from 31% of the SIW examined, but this condition was not found in any other group of bees.

We next tested the possibility that the 4 minute shift to 22^°^C (used to identify SIW from dead bees) could have impacted the hemocyte profile results. For this analysis, 60 active bees were cooled on ice, then divided into two groups. Bees in one group were subjected to a 4 minute temperature shift to 22°C (at which time they began to regain movement) and were then returned to the ice. Bees in the control group were kept on ice without the temperature shift. Hemocyte profiling was then applied to the bees in both groups (n = 30 for each group). Data from Q1, Q2, and Q4 were log-transformed to meet normality, then two-sample t-tests were used for comparison. None of the differences between the groups were statistically significant (Q1 P = 0.66, Q2 P = 0.61, Q3 P = 0.16, Q4 P = 0.81), indicating the temperature shift did not cause significant artifact.

**Fig 8:**
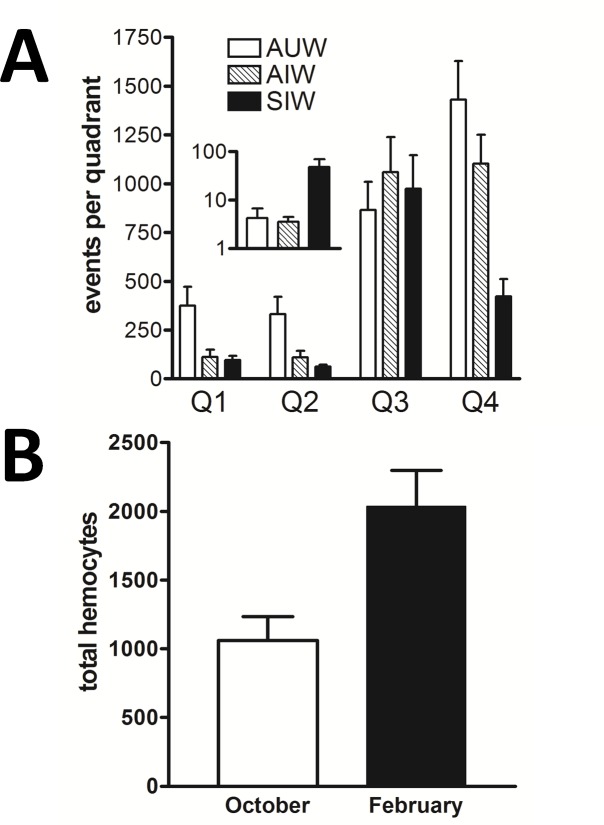
Flow cytometry comparison of hemolymph particulates. A) For each group of live bees examined (AUW, AIW, and SIW) bars represent values from 16 individual bees, for each of the four flow cytometry data quadrants. One-way ANOVA confirmed significant differences (P<0.05) for all three hemocyte populations, by comparing AUW to infected bees. We also confirmed that the concentration of large particulates (primarily pollen grains) was significantly higher (P = 0.008) in the hemolymph of SIW (black bar) compared to both AUW (white bar) and AIW (striped bar), as shown in the inset. B) Actively-clustering AUW were sampled on October 2^nd^ and again the following February 11^th^ and subjected to hemocyte profiling. Total hemocyte numbers showed a significant increase (p = 0.0004, n = 16) during that time, identifying a general increase in hemocyte numbers during winter. Fig B supports the view that the hemocyte decrease observed in Ss1-infected bees was not due to seasonal or temperature effect.

To determine whether the hemocytes in Ss1-infected bees were significantly reduced, we examined the hemolymph particulate occurrence (particles/quadrant) in 16 individual bees representing each of the AUW, AIW, and SIW experimental groups, as shown in Fig 8A. One-way ANOVA identified overall significant (P<0.05) reduction in hemocyte populations of infected bees (confirmed using a Tukey multiple comparison post-test for each of the individual hemocyte populations Q1, Q2, and Q4). In addition, large particulates representing pollen grains were found to be significantly greater (P = 0.008) in SIW relative to AUW and AIW, as shown in the Fig 8A inset. By contrast, the relative abundance of microparticles (Q3) was not significantly different (P = 0.57) when comparing the three groups of bees.

We next examined uninfected bees to determine whether the decrease in the total hemocyte number of SIW and AIW is a seasonal or temperature effect. Active cluster bees were collected and pooled from three different hives on October 2^nd^ and analyzed by hemocyte profiling. The same three hives were again sampled and examined by hemocyte profiling on February 11^th^. Our analyses showed a significant increase (rather than decrease) in total hemocyte numbers (p = 0.0004 n = 16) in normal bees during the time between sampling (Fig 8B). This result suggests the decrease in hemocyte number we found in Ss1-infected bees is not a seasonal or temperature effect.

## Discussion

### A New Bacterium-bee Interaction

We report the isolation of Ss1, a novel bacterium obtained from hemolymph of septic adult worker bees. This organism was also found in honey bee drones, carcasses of worker bees found in winterkilled hives, and about half of *V*. *destructor* examined. Ss1 appears to be distinct from a strain of *S*. *marcescens* causing honey bee brood disease in the Sudan in 1987 [19], because Ss1 was not observed in diseased brood. Ss1 also does not appear to have been identified in previous culture-based detection methods, or by sensitive metagenomic approaches applied to characterize a spectrum of microbes relevant to apiculture [36–38]. Genomic, growth rate, and biochemical properties of Ss1 show clear differences when compared with other *Serratia* examined. Thus, we believe Ss1 has not previously been described.

### Infection of Bees by Ss1

Evidence of infection and associated pathology in honey bees caused by Ss1 was most obvious in SIW and SID. These two groups of live bees were easily identified due to their inability to fly and tendency to separate from uninfected colony members (Figs 1 and 4). These groups were also found to have the highest concentration of Ss1, as shown in Fig 3. We believe presence of Ss1 in bees represents a pathologic condition, and not a manifestation of natural bee senescence. In spite of apparent infection by Ss1, we did not observe infected bees forcibly being removed from hives by patrolling guard or housekeeping bees.

The ability to locate AIW but not SIW during seasons when bees were actively flying raises several considerations. First, this observation suggests that during suitable flying conditions, workers infected by Ss1 abandon the hive while they can still fly. Self-extrication of septic hive members supports hygienic behavior in honey bees, which is believed to limit disease transmission [39]. Second, difficulty locating SIW during warm seasons may be evidence that adult bees do not hatch with symptoms of infection, as when infected by brood pathogens such as deformed wing virus [40]. This could indicate brood does not become infected by Ss1; or that once infected, does not hatch. Third, lack of observed SIW during warm seasons may obscure the presence of Ss1 within the hive. The difficulty of recognizing an exodus of septic bees during warm seasons may temporarily mask disease in a declining colony; a symptom also reported for colony collapse disorder [36].

SIW were found separated from hive members only during cold weather. This observation suggests that the distance that workers are able to separate from the hive when symptomatic may be limited by low temperatures. In contrast, SID were found immediately outside the hive entrance during summer months. This supports hygienic behavior in drones as well as for workers. It also indicates infected drones may be less effective in distancing themselves from the hive when symptomatic. This situation may be due to the fact that SID demonstrated significantly higher (P<0.05) concentrations of Ss1 in the hemolymph than SIW, presumably waiting longer than workers to self-extricate. Vitellogenin has been linked with regulation of immunity and hormonal balance in honey bees [41–43]. Therefore, the particularly high Ss1 concentrations we observed in drones may be related to the fact that unlike worker bees, drones reveal vitellogenin in hemolymph only between 3 and 14 days after hatching [44].

The AIW revealed hemolymph titers of Ss1 averaging less than 1.5 x 10e5 cfu/ml (Fig 3). These infected workers, which comprised less than 3% of colony members, appeared to be functioning normally in spite of sepsis. Identification of these bees supported the notion that transmission of Ss1 continued even when SIW were not observed (during warm seasons). We suggest the AIW represent bees during the early stage of infection by Ss1. These active but infected bees may contribute to bacterial transmission directly to other bees, or indirectly by serving as a reservoir for Ss1 that becomes distributed through phoretic feeding by *V*. *destructor*. The observation that hemolymph of AIW contain a significantly higher (P<0.005) concentration of plasmatocytes and lower (P = 0.032) concentration of pollen grains relative to SIW (Fig 8A) suggests the pathological impact of the Ss1 infection in AIW has not yet fully developed.

### Identification of Unique Sequences in Ss1

We utilized OrthoMCL analysis (http://orthomcl.org/orthomcl/) to examine the Ss1 genome for evidence of genetic features that may have been derived by lateral gene transfer from other bacterial taxa. As a result, 221 genetic elements of Ss1 were identified that did not exist among 41 other members of the *Serratia* genus. A BLAST analysis was performed with these elements, resulting in a number of hits to the NCBI nr database. We used resulting annotations to reveal events of horizontal gene transfer among organisms that presumably share selectable advantages to survive in an insect host. Consequently, we identified 65 genetic elements that do not appear in the 41 queried *Serratia* genomes, including 12 unique sequences of Ss1 that show homology to other bacterial commensals of insects (S2 Dataset). It is not known whether any of these unique genes constitute true virulence factors in honey bees. Additional genes of Ss1 may be responsible for its ability to alternate between the honey bee and *V*. *destructor* as intermediate hosts.

### Detection of Ss1 in *V*. *destructor*

In addition to observing Ss1 in septic honey bees, about half of *V*. *destructor* retrieved from 25 of 33 area hives contained this bacterium as well. Interestingly, the mites we examined either had no detectable Ss1, or had uniformly high concentrations in their extracts (1.7 x 10e9 cfu/ml +/- 4.2 x 10e8 cfu/ml, n = 16) as shown in Fig 3. We noted that some of the freshly-collected, culture-positive *V*. *destructor* demonstrated obvious movement immediately prior to sampling. This observation suggests Ss1 may not kill the mites, which is in contrast to reported acaracide activity of a different strain of *S*. *marcescens* [45].

If *V*. *destructor* are not killed by Ss1, they could transmit this bacterium among members of the hive, and possibly to new colonies when bees undergo drifting [46]. A recent report identified a tendency for hives with higher *V*. *destructor* numbers to be more permissive to drifting bees [47], thus increasing the likelihood of mite-borne disease transmission. The possibility of Ss1 being transferred among honey bees by *V*. *destructor* is consistent with experimental evidence of a pigment-producing strain of *S*. *marcescens* transmission by *V*. *jacobsoni* mites [26]. Although our observation of Ss1 in mites raises the possibility this bacterium is being transmitted among bees during feeding by the mite, additional studies are needed to confirm this situation.

### Pathologic Manifestations in Bees Associated with Ss1

Hemocyte profiling by flow cytometry indicates septic bees experience a loss of cellular immunity (Figs 7 and 8). This suggests infection of honey bee hemolymph by Ss1 suppresses a set of host defense mechanisms. This may compromise the ability of infected bees to limit expansion of the bacterium in the hemolymph. In the lepidopteran insect pathogen *Serratia* sp SCBI, (representing the closest genomic match to Ss1 as shown in Fig 5), a hemolysin was detected in the bacterial culture [48] and cytotoxicity was connected with protease expression [49]. Similar factors in Ss1 may deplete hemocyte populations in hemolymph of infected bees. If confirmed, this result would identify a potential virulence mechanism of Ss1 in honey bees.

We have presented our evidence of declining hemocyte numbers in honey bee workers infected by Ss1 as a possible sign of cellular immune suppression resulting from this infection. However, other mechanisms that reduce circulating hemocyte numbers are also possible. For example, a similar decline in total hemocyte numbers was observed following inoculation of hemolymph of *Manduca sexta* larvae with *Pseudomonas aeruginosa*, *Staphylococcus* aureus and *Micrococcus luteus* [50]. In those studies, hemocytes were suggested to have left circulation following initiation of defensive responses that involved adherence to tissues. Similarly, we cannot exclude the possibility that the reduced number of hemocytes we observed in Ss1-infected bees heralds the onset of the bee’s immune response. Indeed, extracellular host-derived nucleic acids are known to participate in pathogen trapping and microbial killing in hosts ranging from insects to humans [51]. In *Galleria mellonella* larvae, immune complexes containing RNA and DNA have been shown to foster host defense mechanisms including hemolymph clotting and upregulated expression of antimicrobial peptides [52]. By contrast, injection of a red-pigmented strain of *S*. *marcescens* (but not *S*. *aureus* or *E*. *coli*) into *Bombyx mori* larvae resulted in an increase in total circulating hemocytes relative to the vehicle medium control. The ability of that strain of *S*. *marcescens* to increase hemocyte numbers was attributed to a serralysin that contributed to increased bacterial pathogenesis by suppressing hemocyte adhesive properties [53].

We cannot conclude whether circulating hemocytes are reduced in Ss1-infected honey bees due to bacterial-induced immune suppression or hemocyte activation. In either case, the level of sepsis in the SIW and SID was considerable. For example, we frequently measured greater than 1 x 10e10 cfu/ml Ss1 in hemolymph from living drones. By comparison, between 100 and 1000 cfu of human pathogens/ml of blood is frequently fatal in adult humans [54].

During sampling of SIW, hemolymph containing digestive contents was occasionally expelled from the puncture site immediately upon breach of the abdominal cuticle. Careful observation during collection of these samples suggested mixing of digestive contents and hemolymph was not an artifact of sampling, but had occurred prior to collection. Flow cytometry indicated these samples also contained significantly elevated levels of large particulates (Fig 8A, inset), consistent with presence of pollen grains, as confirmed by microscopy (Fig 2B). Our observation suggests that replication of Ss1 in the hemolymph may cause permeabilization of some organ membranes during end-stage infection, resulting in mixed circulatory and digestive contents. This situation may be associated with serralysin-like proteolytic enzymes produced by Ss1, as reported in other *Serratia* members [55,56].

In other SIW, evidence of Ss1 infection in hemolymph without digestive contents (Fig 2A) supports the view that infection of the hemolymph does not require breached digestive system barriers. In addition, bacterial culture of fecal material from bees emerging from winter clusters known to contain Ss1 did not reveal evidence of the bacterium. Our observations collectively support the view that Ss1 infection of honey bees originates in the hemolymph and not in the digestive tract. This scenario of exposure is consistent with inoculation of Ss1 into bee hemolymph during phoretic feeding by *V*. *destructor*.

### Model of Infection by Ss1

Dynamics of infectious agents impacting honey bees have been modeled to reveal surprising complexity in disease transmission within hives [57–59]. Similarly, we expect that the number of bees infected by Ss1 within a colony depends upon several variables. Our data collectively point to the possibility that Ss1 is being transmitted between honey bees by *V*. *destructor* mites. Therefore, potential influences of Ss1 transmission include seasonal changes of hive activity, abundance of *V*. *destructor* in the hive, the percentage of *V*. *destructor* carrying Ss1, the ratio of mites to bees, queen activity, nutritional status and genetic lineage of bees, presence of other infectious agents, and exposure to xenobiotics. The transmission rate of Ss1 within a hive may be particularly high when mite numbers reach maximum levels prior to the onset of colder seasons, as we [35] and others [46] have reported. Therefore, increased loss of bees due to Ss1 in the fall could threaten the critical number of hive members needed to sustain colony thermoregulation [60] and activity through early spring, when brood rearing commences anew.

We linked presence of Ss1 with several symptoms of bee disease. These findings included sepsis in workers and drones, decreased numbers of circulating hemocytes in infected workers, possible permeabilization of organ membranes in workers, and winterkill. In one instance, necropsy of a failed queen revealed 1.1e9 cfu/ml of Ss1 in the hemocoel extract. By contrast, we have also noted that colonies containing Ss1 could persist for over one year, as SIW, SID, AIW, and culture-positive *V*. *destructor* were all observed. Therefore, attrition of colony members due to infection by Ss1 may be offset when brood rearing is active, creating covert hive disease.

### Question on Causation

We attempted to apply Koch’s Postulates to confirm the pathogenic potential of Ss1 in live honey bees. This was done by inoculating hemolymph directly in live bees to determine whether Ss1 could experimentally replicate natural infection and disease in test subjects. However, because of high mortality in honey bees from cold-anesthetization, we were unable to utilize experimental methods to achieve this goal. Therefore, our results contrast a recent report [61] utilizing cold-anesthetized honey bees as an infection model of bacterial sepsis. At this time, we are not aware of other reports that describe experimental injection of adult worker honey bee hemolymph to establish sepsis caused by honey bee pathogens. Despite association of Ss1 with disease, death, and winterkill in honey bees, our studies do not provide causal evidence for Ss1 in hive failure. Consequently, we cannot rule out other factors in associated bee maladies.

### Conclusion

We report the isolation and characterization of the novel Ss1 bacterium from the hemolymph of diseased honey bees presenting evidence of decreased cellular immunity. Ss1 was also isolated from a subset of *V*. *destructor* cohabitating with septic bees. We linked this infection to wintertime failure of hives in western Wisconsin, but did not establish causation of hive failure to this bacterium. Additional studies are needed to elucidate the transmission and impact of Ss1 within the complex environment of the hive.

## Materials and Methods

### Reagents and Honey Bee Acquisition

Studies did not involve endangered or protected species. After obtaining permission from the owners of the private apiaries in Western Wisconsin, 3,219 total bees and 1,259 *V*. *destructor* mites were collected for analyses. All reagents and chemicals utilized in this study were purchased from either Fisher Scientific (Waltham, MA, USA) or Sigma Aldrich (St. Louis, MO, USA). Colonies of honey bees involved in this study were housed in standard 10-frame, deep-body Langstroth hives, with one additional top-bar hive system (https://www.beepods.com/). Our studies included Carniolan and Italian honey bees, without obvious differences between bee strains. Sampling of honey bees from hives was carried out as described [35], and involved standard methods in honey bee research [62].

### Microbe Cultivation and Enumeration

For honey bee hemolymph analyses, samples were obtained from live cold-anesthetized bees [35], with some modifications as described below. Individual hemolymph samples were sometimes subjected to both flow cytometry and culture analysis by first puncturing the dorsal aspect of the bee’s abdominal cuticle with a sterile 26-guage hypodermic needle, then 2 μl of hemolymph was collected in a sterile micropipette. The sample was diluted to 200 μl in flow cytometry buffer prepared as described [35]. An aliquot of the diluted hemolymph was immediately obtained for serial dilution and culture analysis, while the remainder was examined by flow cytometry. The aliquot for culture was subjected to serial dilution in sterile LB broth and spread on LB agar plates, then incubated 3 days at 22°C. Concentrations of Ss1 in the hemolymph were calculated from the dilution factor, volume cultured, and plate counts. A commercially available strain of *S*. *marcescens* (Presque Isle Cultures, catalog #361) was used for comparison of colony phenotype and growth dynamics in liquid LB broth.

For culture analysis of dead bees from winterkilled hives, bee carcasses were recovered within 21 days following hive failure, when daily temperatures remained below 0°C. Samples of winterkilled bees were sometimes stored at -20°C for up to two weeks prior to culture analysis. We confirmed that bees stored frozen at -20°C retain culture positivity of Ss1 for at least 12 months. Samples from bee carcasses were collected as for hemolymph from live bees, except that 10 μl of sterile water was first introduced into the hemocoel to suspend microorganisms when present. Then 3 μl of the hemocoel extract was retrieved for bacterial culture as described above.

Fecal material from living bees exiting hives (that also contained Ss1) was cultured to determine if the bacterium was carried in the digestive tract. During warm early spring days when bees began to fly after weeks of confinement, fecal material expelled by bees during flight was collected on paper sheets placed on the ground around the hive. Samples were collected on swabs which were used to inoculate LB agar plates for culture analysis as described above.

*V*. *destructor* were obtained for culture analysis from adhesive mite trap sheets placed under screen bottom boards of hives. Sheets were removed from hives within 72 hours to ensure recovery of fresh mites. Individual mites attached to the adhesive surface were crushed in place by gentle tapping with a sterile pipette tip. Contents of the mites were then suspended in 3 μl sterile water and examined by serial dilution and culture analysis.

### Microbe Characterization

#### Biochemical Reactions

Biochemical analyses of Ss1 involved both use of standard carbohydrate utilization reactions manufactured by Fisher Scientific, as well as the Analytical Profile Index 20E (bioMerieux, Durham, NC) test system. Esculin hydrolysis was evaluated using bile-esculin agar (Remel #R01190, Lenexa, KS). Urease production was determined using urease broth (Oxoid #CM0071, Basingstoke, Hampshire, England). DNase reactions were determined in DNase agar with methyl green (Difco #222020, Voigt Global Distributing, Lawrence KS). Unless otherwise indicated, incubation of reactions for biochemical analyses was performed at 28°C and incubated for 7 days before being regarded as negative.

#### Liquid Growth Characterization

Turbidity readings were used to compare growth characteristics of Ss1 with a commercially-available control strain of *S*. *marcescens* (Presque Isle Cultures, catalog #361) in liquid LB medium. Cultures were first prepared by inoculating two ml of LB broth with single colonies of the respective strains of the bacteria and allowing them to grow without shaking at 22°C until the optical density reached 0.06 at 600 nm. A sample of each culture was diluted 40-fold in fresh LB medium and 0.2 ml aliquots of the diluted samples were distributed to a sterile 96-well microtiter plate. A Molecular Devices Spectramax Plus 384 96-well plate reader was used to measure the turbidity of wells at 600 nm every 15 minutes without mixing for 20 hours at 22°C. The raw absorbance data were then examined to determine the maximum rate of turbidity increase (Vmax) as indicated by the Molecular Devices SoftMax Pro ver. 5.4 software, and the final optical density of the cultures after 20 hours of incubation. Replicate samples were prepared to represent the sterile LB broth medium as a reference, and to measure the growth of Ss1 compared with the control *S*. *marcescens* strain.

#### Genetic Characterization

A pure culture of Ss1 was grown at 22^°^C for 36 hours in liquid LB medium and subjected to 16S rRNA sequence analysis at the University of Wisconsin-Madison Biotechnology Center. The 480 base pair DNA sequence corresponding to the V3-V4 region 16S rRNA was determined and examined using the Ribosomal Database Project Naïve Bayesian Classifier at Michigan State University [63]. This analysis indicated the organism belongs to the *Serratia* genus within the *Enterobacteriaceae* family. Whole-genome nucleotide sequence analysis was then carried out (data available through Genbank). The entire 1541 base pair 16S rRNA sequence of Ss1 was then used to probe nucleotide databases through the NCBI nucleotide BLAST function (http://blast.ncbi.nlm.nih.gov/Blast.cgi).

The Ss1 genomic sequence was compared with whole assembled genomes from 41 other *Serratia* organisms obtained from the ASAP database (https://asap.genetics.wisc.edu/asap/home.php) at the University of Wisconsin-Madison. Genome comparisons were then made with both the nucleotide sequence and the deduced amino acid sequences [21,22]. ANI and AAI scores were calculated using custom scripts based on those provided online [64]. The data were transformed to a distance matrix with the formula (100-%ID)/100 and analyzed with the Fitch program in the Phylip suite of tools version 3.7a (http://evolution.gs.washington.edu/phylip/doc/main.html). The resulting tree was processed by Archaeopteryx [65] for presentation.

The OrthoMCL algorithm [33] was applied to identify orthologous proteins in this group of 42 *Serratia* genomes. This allowed a determination of features that are shared among *Serratia* isolates in addition to others that are unique to any particular organism in this set. A BLAST analysis of features specific to strain Ss1 was performed against the NCBI nr database and the genus of the best hit was recorded as the origin of the sequence.

### Flow Cytometry and Microscopy

Hemolymph diluted in buffer as indicated above was incubated at 37°C for 5 minutes then examined immediately by flow cytometry as described [35]. The FSC collection threshold value of 25 was applied to increase representation of hemocytes in data analyses. Using this setting, some particles (debris, bacteria, and smaller microparticles) were not detected. Flow cytometry events were also gated in the FSC versus SSC plot to focus fluorescence evaluation on hemocyte and microparticle populations. Red and green fluorescence plots were used to evaluate particle binding by PI and WGA-FITC, respectively. For direct microscopy of hemolymph samples, wet mounts were prepared by placing 0.5 μl of undiluted hemolymph between a glass slide and coverslip. Photomicrographs of undiluted and unstained hemolymph samples from septic bees were obtained using differential interference contrast imaging at 1000 X total magnification as described [35].

### Statistical Analyses

A Shapiro-Wilk test for normal distribution and Levene’s test for homogeneity of variance were applied to data sets before examination by ANOVA. If necessary, log transformation of data was applied to meet the assumptions of ANOVA. If the overall P value was below 0.05, pairwise comparisons were made in a Tukey analysis. Mean values are given as ± standard error of the mean. In all analyses, P values of <0.05 were considered significant. Statistical analyses were performed using R software (version 3.0.2).

## Supporting Information

S1 Dataset Whole-genome identity scores for comparisons of Ss1 to genomes of other *S*. *marcescens* strains. The degree of homology reflecting pair-wise comparison between genomes (as described in the Methods) is expressed in values of percent identity for the organisms indicated. Data for both ANI and AAI are shown on the respective spreadsheet tabs. Values involving comparison to Ss1 are shown in yellow highlighting.
(XLSX)

S2 Dataset Ss1-specific features identified within the coding sequences of the genome. The OrthoMCL [33] dataset was used to reveal nucleotide sequences in the Ss1 genome that encode proteins not found in the 41 other *Serratia* genomes included in the S2 Fig. The columns include data from ASAP (Feature ID, annotated product, and feature length), as well as data from the GenBank entries for the best BLAST hits, such as genome of origin, NCBI accession number, Expect values (E-values) of the BLAST hit to the GenBank feature, and the product as annotated in GenBank. E-values indicate the number of hits that can be anticipated by chance, where values closer to zero identify the more significant matches. The ASAP feature length gives the deduced protein size in the number of amino acid residues.
(XLSX)

S1 Fig The genomic nucleotide sequence representing the complete 16S rRNA of Ss1. This 1541 base-pair DNA sequence encoding the 16S rRNA of Ss1 was determined from the whole-genome nucleotide sequence. The nucleotide sequence was found to have a 99–100% identity to several other strains of *S*. *marcescens*, including the *S*. *marcescens* type strain, ATCC 13880.
(TIF)

S2 Fig Dendrogram of 42 *Serratia* clones based on Amino Acid Identity (AAI). The whole genome nucleotide sequence of Ss1 was used to extrapolate the encoded proteome, which was then compared using AAI analysis to 41 other sequenced strains of *Serratia* for evidence of relatedness at the level of amino acid sequence. The distance scale values comparing the organisms in pair-wise matches are included in the Supporting S1 Dataset.
(TIF)

S3 Fig DNase activity of Ss1 compared with other honey bee commensals. Various microbes were cultivated from the surface of normal honey bees, propagated in pure culture, and then inoculated to the seven unmarked sections on the 10 cm plate containing DNase agar with methyl green. The section designated by an asterisk was inoculated with a pure culture of Ss1. The plate was then incubated at 22°C for three days. Resulting DNase activity was shown only around the growth of Ss1, which was indicated by clearing of the blue color. This result supports the utility of this culture medium in screening for Ss1.
(TIF)
