# Supplementary figures and images for: Sepsis and Hemocyte Loss in Honey Bees (Apis mellifera) Infected with Serratia marcescens Strain Sicaria
Source: PLoS One. 2016 Dec 21;11(12):e0167752. doi: 10.1371/journal.pone.0167752 (PMC5176276; doi:10.1371/journal.pone.0167752)

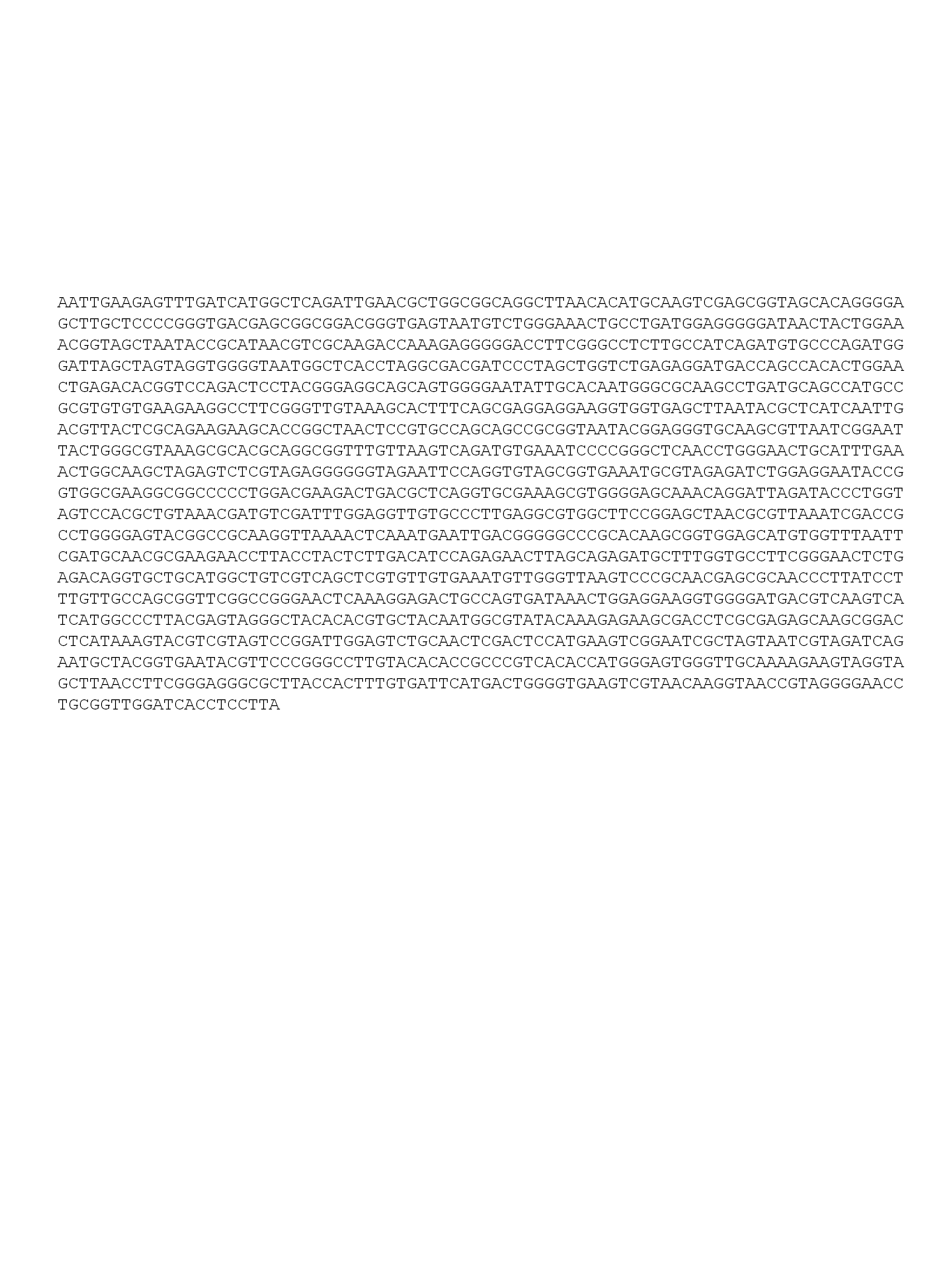

Supplement: S1 Fig — This 1541 base-pair DNA sequence encoding the 16S rRNA of Ss1 was determined from the whole-genome nucleotide sequence. The nucleotide sequence was found to have a 99–100% identity to several other strains of S. marcescens, including the S. marcescens type strain, ATCC 13880. (TIF) [file pone.0167752.s003.tif]

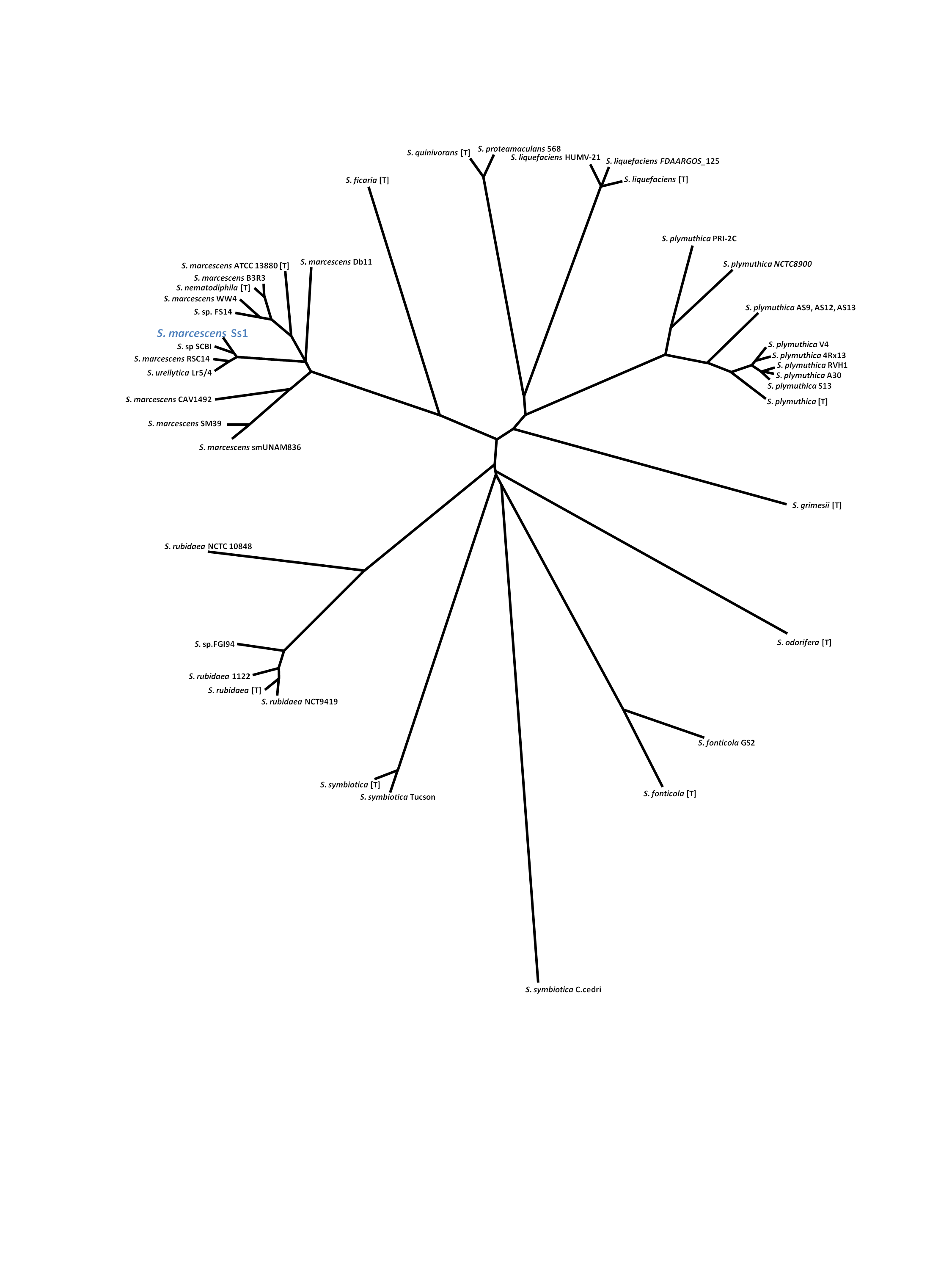

Supplement: S2 Fig — The whole genome nucleotide sequence of Ss1 was used to extrapolate the encoded proteome, which was then compared using AAI analysis to 41 other sequenced strains of Serratia for evidence of relatedness at the level of amino acid sequence. The distance scale values comparing the organisms in pair-wise matches are included in the Supporting S1 Dataset. (TIF) [file pone.0167752.s004.tif]

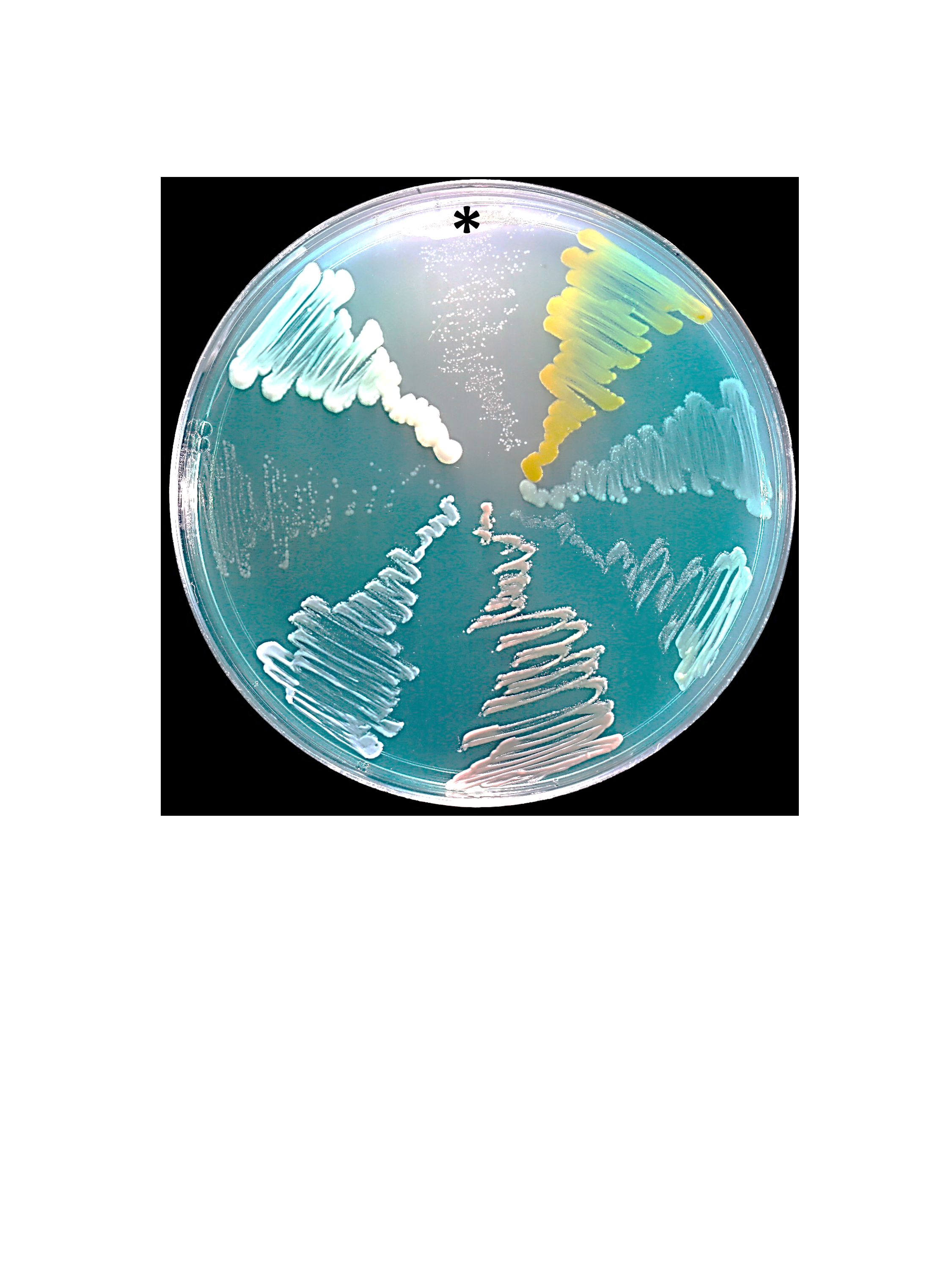

Supplement: S3 Fig — Various microbes were cultivated from the surface of normal honey bees, propagated in pure culture, and then inoculated to the seven unmarked sections on the 10 cm plate containing DNase agar with methyl green. The section designated by an asterisk was inoculated with a pure culture of Ss1. The plate was then incubated at 22°C for three days. Resulting DNase activity was shown only around the growth of Ss1, which was indicated by clearing of the blue color. This result supports the utility of this culture medium in screening for Ss1. (TIF) [file pone.0167752.s005.tif]
